# Intratympanic Methylprednisolone as Rescue Therapy in Sudden Sensorineural Hearing Loss

**DOI:** 10.1590/S1808-86942010000400015

**Published:** 2015-10-19

**Authors:** Igor Teixeira Raymundo, Fayez Bahmad, Jairo Barros Filho, Thaís Gonçalves Pinheiro, Nilda Agostinho Maia, Carlos Augusto Oliveira

**Affiliations:** a Medical resident of the Medical Residency Program in Otorhinolaryngology and Head & Neck Surgery, Brasilia University Hospital (Hospital Universitário de Brasília - HUB), University of Brasilia (UnB); b Doctoral degree in the Medical Science Program, UnB Medical School. Medical teacher in the Otorhinolaryngology and Head & Neck Surgery Program, HUB, UnB; c Medical resident of the Programa de Residência Médica do Hospital Universitário de Brasília - HUB-UnB; d Medical resident of the Programa de Residência Médica em Otorrinolaringologia e Cirurgia de Cabeça e Pescoço do Hospital Universitário de Brasília - HUB-UnB; e Speech therapist specialized in audiology. Head of the Speech Therapy Department, HUB, UnB Medical School; f Post-Doctoral degree at the Harvard Medical School, Boston, MA, EUA. Head of the Otorhinolaryngology and Head & Neck Surgery Department, HUB, UnB. Full professor of the Otorhinolaryngology Discipline, UnB Medical School

**Keywords:** hearing loss, sudden, hearing loss

## Abstract

Treatment in sudden sensorineural hearing loss is a contentious issue, today, oral steroids are the most common choice and considered the best treatment option, but the use of intratympanic steroids has become an attractive alternative, especially in cases when systemic therapy fails, or to avoid the side effects of the systemic use of steroids.

**Aim:**

To describe the results of intratympanic methylprednisolone in idiopathic sudden sensorineural hearing loss after failure of oral prednisolone.

**Methods:**

In a prospective study fourteen patients with idiopathic sudden sensorineural hearing loss were treated with intratympanic methylprednisolone after failing in the treatment with systemic steroids. Pretreatment and post-treatment audiometric evaluations including pure tone average (PTA) and speech reception thresholds (SRT) were analyzed.

**Results:**

Ten from 14 patients treated with intra-tympanic methylprednisolone presented with hearing recovery > 20 dB in PTA or 20% in SRT.

**Conclusion:**

Three intratympanic injections of methylprednisolone improved pure-tone average or speech discrimination scores for a subset of sudden hearing loss subjects that failed to benefit from oral steroids.

## INTRODUCTION

Sudden hearing loss (SHL) may be defined as hearing loss of 30dB or more that occurs within three days, and affects three or more frequencies.^1^ SHL affects from 5 to 20 persons per 100,000 every year, about 4,000 new cases annually in the United States of America.^2^ Hearing loss is nearly always unilateral and is commonly associated with tinnitus and auricular fullness. The true incidence of SHL in Brazil is probably underestimated because many patients recover their hearing within a few days and generally do not seek medical care.

Although this entity is not one of the most common causes of deafness, interest in studying it remains low, probably due to its reversible nature in most cases.^3^

The etiology, natural history, and treatment of this entity have been a matter of discussion for several years. The actual number of patients that recover spontaneously from SHL without medical treatment remains unknown. The elevated rate of spontaneous recovery - over 65% in some studies - also confounds the reviews on the efficacy of a single drug or a new intervention. Any intervention should have improvement rates higher than a 65% assumed spontaneous recovery rate without therapy. The treatment of SHL patients varies among otology units, and there is no universally accepted protocol.

With no specific etiology and a short time period within which to provide effective therapy, generally therapy is but a “ shot in the dark,” which includes several approaches based on hypothetical etiologies. The therapeutic window is short, which makes it difficult to assess the efficacy of detailed studies and individual agents. A paucity of studies limiting the time frame within which maximum recovery may occur - from days^5^^,^^6^ to months^7^^,^^8^ - also complicates any evaluation of efficacy versus the natural history of the disease. Notwithstanding the high spontaneous recovery rates presented in some papers, our experience - and that of most authors - is that recovery of hearing is poor in patients that do not benefit from systemic corticoid therapy.^9^^,^^10^

Several treatment protocols have been proposed for treating SHL. Corticosteroids, antiviral agents, anticoagulants, vasodilators, anti-inflammatory drugs, and other approaches have been suggested; there are reports of some benefit from most of these therapies. At present, the most commonly accepted treatment is systemic corticosteroid therapy. Although its efficacy has been demonstrated in prospective, randomized, double-blind, placebo-controlled studies,^1^^,^^11^ other studies still question the efficacy of systemic corticosteroids for the treatment of SHL.^2^^,^^4^^,^^8^

The early and late complications of systemic corticosteroid therapy are not rare and are well-known to otorhinolaryngologists; this has led to investigations of topical therapies in the inner ear for its conditions, including SHL. Intratympanic corticosteroid therapy may potentially provide organ-specific treatment, with high doses applied over the round window membrane, thereby avoiding the adverse effects of systemic corticosteroid therapy. As with other proposed treatments, the efficacy of intratympanic therapy for SHL needs to be determined; several studies have demonstrated favorable results, even after systemic therapy had failed.^9^^,^^12^, ^13^, ^14^, ^15^, ^16^, ^17^, ^18^, ^19^, ^20^, ^21^

## INTRATYMPANIC THERAPY

Itoh first reported using intratympanic corticosteroids for the treatment of inner ear disease in patients with Ménière's disease in 1991.^22^ Silverstein (1996) first reported using intratympanic corticosteroid therapy for SHL. Other authors have also described the use of intratympanic corticosteroids for the treatment of SHL.^9^^,^^12^, ^13^, ^14^, ^15^, ^16^, ^17^, ^18^, ^19^, ^20^, ^21^^,^^23^, ^24^, ^25^, ^26^, ^27^

Although its efficacy alone has not been proved definitively, intratympanic corticosteroid therapy for SHL is becoming more widely used. The variability among treatment protocols for SHL also applies to intratympanic corticosteroid therapy. Use of intratympanic corticosteroids has given rise to three main protocols for the treatment of SHL:

• Primary Therapy - as the first treatment for SHL, without systemic corticosteroids;
• Adjuvant Therapy - concomitantly with systemic corticosteroids, and
• Rescue therapy - started after systemic corticosteroid therapy has failed.

The fact that stimulated intratympanic corticosteroids without systemic corticosteroid therapy was the existence of a group of patients that did not tolerate the systemic side effects of high dose systemic corticosteroid therapy, such as diabetic patients or those with elevated blood pressure that was difficult to control.^13^^,^^15^^,^^16^

Most studies about intratympanic corticosteroids for treating SHL report having attempted this mode of therapy after systemic corticosteroid therapy has failed.^9^^,^^12^, ^13^, ^14^, ^15^, ^16^, ^17^, ^18^, ^19^, ^20^, ^21^^,^^25^, ^26^, ^27^ Two studies evaluated the effects of intratympanic corticosteroids as adjuvant therapy to systemic corticosteroids in SHL patients.^23^^,^^24^ There are several advantages of using intratympanic corticosteroids (see Frame I). The procedure is well tolerated and relatively easy to perform. Most patients understand the concept of intratympanic therapy and accept it, since it may be administered in an outpatient setting under local (topical) anesthesia.

**FRAME I:** Transtympanic Methylprednisolone for the Treatment of Sudden Hearing Loss

**Advantages**
Outpatient procedure
Easily administered
Administered soon after the diagnosis
Relatively painless
Possible use in patients in which corticosteroids are contraindicated (e.g.: immune suppression, HIV, tuberculosis, diabetes)
High drug concentration when administered directly on the affected ear
Adverse effects are rare
**Disadvantages/complications**
Tympanic membrane perforation
Pain
Otitis media
Vertigo (generally temporary)
Hearing loss

As opposed to systemic treatment, intratympanic therapy acts specifically on the affected ear. Other adverse effects of systemic corticosteroid therapy, besides glucose intolerance and avascular hip necrosis, are insomnia, irritability, gastritis, and altered humor, which may be avoided with intratympanic therapy. The main disadvantage of intratympanic corticosteroid use is lack of proof about its superiority compared to systemic corticosteroid therapy. Other disadvantages are pain, vertigo, and the rare possibility of tympanic membrane perforation and serous otitis media.

The techniques for intratympanic corticosteroid perfusion in the middle ear differ in several aspects, such as the type of corticosteroid to be applied. Dexamethasone is the most common steroid used intratympanically,^14^^,^^15^^,^^18^, ^19^, ^20^, ^21^^,^^24^^,^^25^ followed by methylprednisolone.^9^^,^^13^^,^^15^^,^^16^^,^^17^^,^^23^^,^^26^^,^^27^ Reports in the literature differ about the concentration of solutions (dexamethasone: 2-4 mg/mL^14^^,^^20^ to 25 mg/mL;^15^ methylprednisolone 32 mg/mL^23^ to 62.5 mg/mL).^9^^,^^16^^,^^17^ The amount injected in the middle ear in published papers ranges from 0.3 to 0.5 mL, which is approximately the middle ear volume. Administration modes also differ: transtympanic needle injection,^9^^,^^13^^,^^15^^,^^19^^,^^20^^,^^17^^,^^24^, ^25^, ^26^, ^27^ myringotomy,^13^^,^^14^ myringotomy with a ventilation tube,^12^^,^^23^ myringotomy with a special perfusion needle (Micromedics, Eagan, MN),^18^^,^^21^ and implantable infusion pumps in the middle ear (Round Window m-Cath; Durect Corp., Cupertino, CA) for continuous steroid release.^16^^,^^17^^,^^21^ The duration of treatment, the interval between injections, and the number of injections also differs among authors, ranging from a single dose to weekly transtympanic injections,^9^^,^^12^^,^^14^^,^^15^^,^^18^^,^^20^^,^^23^ steroid solutions as drops applied by patients during several weeks,^18^^,^^21^ transtympanic injections several times a week,^19^^,^^23^^,^^25^^,^^26^ or implantable infusion pumps.^16^^,^^17^^,^^21^

Reported complications are rare, and include pain,^13^ vertigo,^13^^,^^16^^,^^17^^,^^20^^,^^21^ otitis media,^13^ perforated tympanic membrane,^9^^,^^21^ acne,^20^ dysgeusia,^21^ chronic otitis media,^21^ and subsequent hearing loss.^16^^,^^21^ The purpose of this study was to review the experience of using 40mg/ml intratympanic methylprednisolone administered in three injections at 48 hour intervals for SHL patients in which systemic corticosteroid therapy failed to improve hearing. Special attention was given to an evaluation of efficacy, safety, and the correlation with the beginning moment of treatment.

## MATERIALS AND METHODS

From January to December 2008, 64 patients with a diagnosis of sudden sensorineural hearing loss were seen at the otorhinolaryngology outpatient and emergency unit of the University Hospital.

This study was approved by the institutional review board of the Medical School and the University Hospital in which it was carried out. All patients received information about the risks and expectations of therapy and signed a free informed consent form. A detailed clinical history was taken, followed by an otoneurological examination, and an initial audiological assessment by tonal and vocal audiometry, impedance testing, speech recognition rate (SRR), laboratory tests, and an image exam (magnetic resonance imaging with contrast of the inner auditory canal). All patients were treated with systemic therapy according to the local protocol (1mg/kg/day prednisolone for 10 days, followed by decreasing doses thereafter). No patient was given acyclovir antiviral therapy.

Next, salvage therapy with intratympanic methylprednisolone was offered to 64 patients with a diagnosis of SHL, after systemic therapy failed and no improvements were demonstrated audiometrically. Fourteen patients agreed to undergo rescue therapy and signed the free informed consent form to be included in this study. Below are the details of inclusion criteria, the audiometric assessment, application technique, analysis of recovery, and statistical analysis.

### Inclusion Criteria

There were 14 patients included from an original group of 64 candidate patients with sudden sensorineural hearing loss; the 14 patients did not benefit from oral prednisolone (1mg/kg/day) for the treatment of sudden sensorineural hearing loss, that is, they did not improve as shown below under the item analysis of recovery.

Patients that did not meet the inclusion criteria described in Frame 2 were excluded from the study; these were patients with incomplete clinical data or follow-up, inadequate audiometry, or that had undergone more than three injections or that were given different concentrations from those standardized in this study (40mg/ml) were excluded. All patients with fluctuating hearing loss or suspected Ménière's disease were also excluded from this study.

**FRAME 2:** Inclusion Criteria

• Sudden sensorineural hearing loss of at least 30 dB in three frequencies within three days
• No benefits from oral 1mg/kg/day prednisolone therapy during 10 days (unilateral) or 30 days (bilateral)
• At least one audiometric test before and after oral therapy and another before and after intratympanic treatment
• Undergoing three intratympanic 40mg/ml methylprednisolone injections on alternate days
• No previous otological surgery
• No history of Ménière's disease or fluctuating hearing before or after treatments (oral or intratympanic)
• No signs of acute or chronic otitis media

### Audiometric Analysis

All patients underwent tonal and vocal audiometry and speech recognition rate analysis done by audiologists before and after treatment. The tritonal mean was calculated based on pure tone audiometry (PTA) at 0.5, 1.0 and 2.0 KHz. The speech recognition rate (SRR) was based on the percentage of correct answers for monosyllables.

### Technique

Prior to any procedure, patients were oriented as to the risks and expectations about the procedure and signed a free informed consent form. EMLA cream (AstraZeneca, Wilmington DE) was applied for topical anesthesia. EMLA cream was placed in the outer ear canal and the tympanic membrane and left for 30 to 45 minutes, after which it was removed. Next, the patient's head was placed at 45° towards the unaffected ear. A 40 mg/mL methylprednisolone solution was warmed to body temperature in a water bath. About 0.3 to 0.5 mL of the solution was injected into the middle ear; two orifices were made with the drug application needle (Gelco N.22), one immediately below the umbus (where the drug will be administered) and another on the postero-superior region (vent hole). No ventilation tubes were needed.

After intratympanic application of the steroid, the patient remained in the supine position and cervical rotation at 45° for 30 minutes to maximize exposure of the round window membrane to the solution. A second injection was done if there was any possibility that the first injection was not adequate. Patients were asked to avoid water in the treated ear for at least two weeks.

### Definition of Improvement (analysis of recovery)

The criteria for defining successful recovery after therapy vary in the literature on intratympanic therapy. A 20 dB improvement at 0.5, 1 and 2KHz, or a 20% improvement in discrimination was enough to consider the intervention as successful.

Failure of oral prednisolone therapy was absence of improvement, as just described, after 14 days of treatment.

### Statistical Analysis

The SPSS® software for Windows® v. 13.0 (2004) was used for the statistical analysis, which consisted of a sample description: sex, possible cause of the clinical picture, most relevant comorbidities, other symptoms, age, time elapsed before starting oral corticosteroid therapy (OCT), time elapsed before starting intratympanic corticosteroids (ITC). Further analysis consisted of analyzing the tritonal mean (PTA) before treatment, after treatment with OCT and after ITC. This measurement is the mean value of audiometry at 500, 1000 and 2000 Hz, and it was used to compare our results with those in the literature. A 20 dB audiometric decrease for PTA indicated a clinical improvement.

Bivariate correlations were also tested (Pearson's correlation coefficient) between the initial loss in PTA (dB) and improvement in PTA after OCT and ITC, and the time elapsed until starting ITC and improvement in PTA (dB).

Finally, mean audiometric values at each frequency (250, 500, 1000, 2000, 3000, 4000, 6000 and 8000 Hz) and the speech recognition rates (SRR) before and after OCT and after ITC were compared using an analysis of variance model for repeated measures, with the purpose of establishing the effectiveness of therapy. The requisite sphericity requisite was met for all analyses (Mauchly's W > 0.744, p>0.170 for all analyses), such that no correction method was needed to correct degrees of freedom.

The Bonferroni method was applied to correct the level of statistical significance for multiple comparisons. The statistical significance value (p-value) was 5% (p<0.05) throughout.

## RESULTS

### Patients

Table 1, Table 2 show the descriptive data of the study sample. After applying inclusion and exclusion criteria, 14 patients remained in the study. There were 8 female (57.14%) and 6 male patients (42.86%).

**Table 1:** 

Description of the sample	% (n)
Sex	
Female	57,14 (8)
Male	42,86 (6)
Cause	
ISSHL	85,7 (12)
Viral labyrinthitis	14,3 (2)
Comorbidity*	
None	64,3 (9)
DM2	28,6 (4)
SAH	21,3 (3)
Glaucoma	14,3 (2)
Other symptoms	
Tinnitus	85,7 (12)
Vertigo	14,3 (2)
URI symptoms	21,3 (3)
Otalgia	7,1 (1)
Improved clinical picture**	
After OCT	0
After ITC	71,4 (10)

OCT: Oral Corticosteroid Therapy

ITC: Intratympanic Corticosteroid Therapy

**Table 2:** 

Description of sample	mean (EP)	median
Age	43,79 (4,46)	42
Start of OCT (h)	94,29 (28,69)	72
Start of ITC (d)	16,86 (1,29)	15
PTA initial (dB)	71,67 (7,63)	78,33
PTA after OCT (dB)	65,71 (8,38)	68,33
PTA after ITC (dB)	40,95 (8,31)	41,67

h: hours; d: days

OCT: Oral Corticosteroid Therapy

ITC: Intratympanic Corticosteroid Therapy

PTA: Tritonal Mean

Image and laboratory exams revealed viral labyrinthitis in 2 of 14; the other 12 patients remained without a diagnosis after careful investigation. Their final diagnosis was idiopathic sudden sensorineural hearing loss (ISSHL).

Comorbidities included systemic arterial hypertension (SAH) or type 2 diabetes mellitus (DM), which were present in 5 patients, of which three patients had SAH and type 2 DM, and two had a history of increased intraocular pressure. Hearing did not improve in any of these 14 patients with oral prednisolone therapy. The recovery rate after Intratympanic therapy was 71.4%, corresponding to 10 of 14 treated patients (see Table 1).

It is worth noting that all patients were treated systemically with prednisolone, even those with type 2 DM or SAH, in which a cardiologist and an endocrinologist approved the treatment.

The mean age was 43.79 years, ranging from 25 to 72 years. The mean age of female patients was 38.37, and the mean age of male patients was 48.16 years.

### Recovery

**Figure 1:**
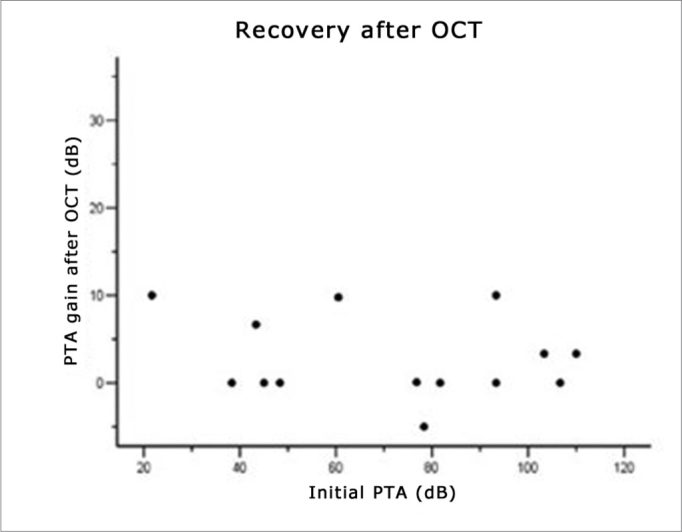
Recovery after oral corticosteroid therapy.

**Figure 2:**
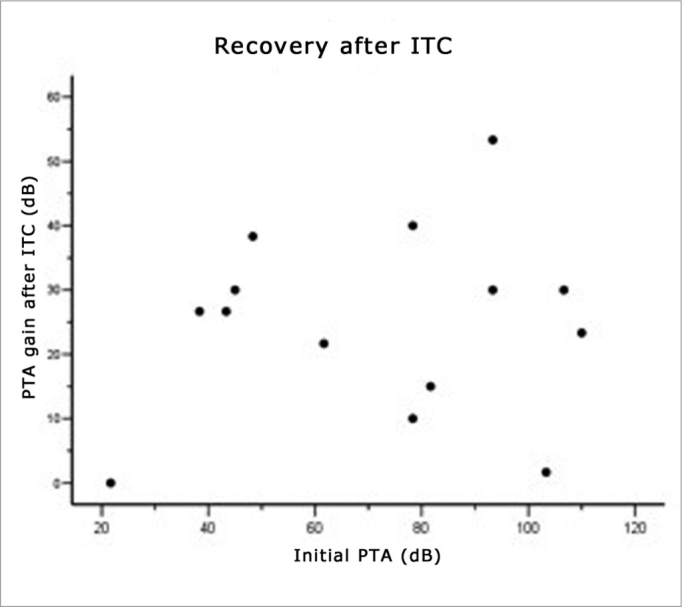
Recovery ratio following intratympanic corticosteroid therapy and the initial tritonal mean.

The initial tritonal mean (PTA) of 14 patients that did not meet the improvement criteria after oral therapy was 71.63 dB (median – 78.33); after fourteen days of oral therapy, it was 65.71 dB (median – 68.33) (see Fig. 1). One week after intratympanic therapy, the tritonal mean was 40.95 dB (median – 41.67) (see Table 2 and Fig. 2).

Improved tritonal mean or SRR was seen in 71.40% of patients (n = 10); the mean gain among these 10 patients was 27.33 dB, ranging from 20 to 55 dB. Four patients did not improve according to our criteria; three of these had respectively a 5, 10 and 15 dB improvement of their tritonal means, and one patient had no auditory change after intratympanic therapy (Fig. 2).

### Age Factor

Age-related recovery was analyzed for each patient. Pearson's test showed that there were no statistically significant correlations between age and improvement after ITC (r=0.103, p=0.726).

One patient among the 14 subjects was aged over 60 years; this patient was aged 72 years and had SAH and type 2 DM, a radiological and laboratory diagnosis of infectious viral labyrinthitis, and did not improve according to the abovementioned criteria. The other three patients that did not improve after intratympanic therapy were aged from 25 to 55 years. The two patients that had a radiological and laboratory diagnosis of infectious labyrinthitis did not benefit from intratympanic therapy.

### Recovery Related With Other Symptoms

Tinnitus was present in 12 patients (85.7%); the recovery rate in these patients was similar to those without this complaint. Vertigo was present in 2 patients (14.3%) and their recovery rate was 0%.

### Situation of the Contralateral Ear

Normal hearing in the contralateral ear was present in 92.9% of patients. The recovery rate in this group was 70%. One patient (7.1%) had abnormal hearing in the contralateral ear, possibly because of presbyacusis, as this patient was aged 72 years and did not improve with therapy.

### Recovery Related With the Time of Onset of Symptoms and Therapy

**Figure 3:**
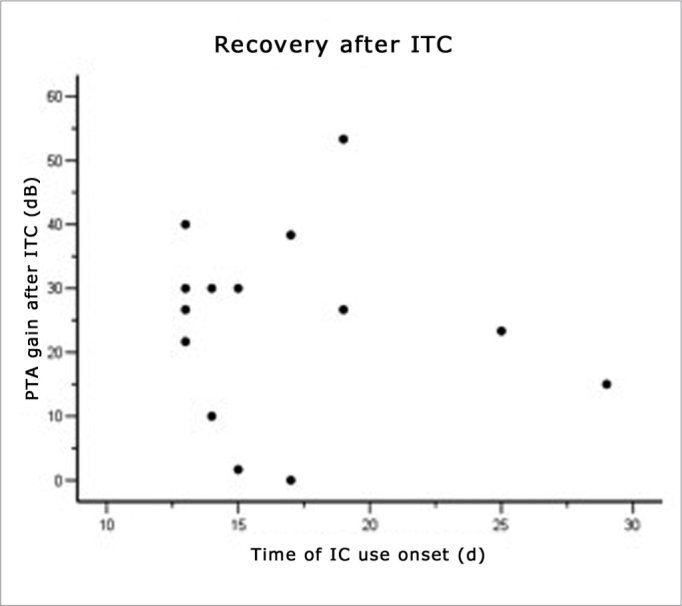
Recovery ratio following intratympanic corticosteroid therapy and the time elapsed before starting corticosteroid therapy.

(See Figure 1, Figure 2, Figure 3, and Table 3) The mean time elapsed before starting OCT in the 14 patients was 94.29 hours, that is, between the third and fourth day after the onset of symptoms (Fig. 1). The time elapsed before starting intratympanic therapy was 16.86 days after the onset of symptoms (Fig. 3). Patients that started therapy soon after failures of systemic therapy was detected had an evident advantage. Patients that started Intratympanic therapy after the third week had a lower success rate compared to the group treated from 14 to 21 days (see Table 3).

**Table 3:** 

Recovery related to the time elapsed before therapy was started and the onset of symptoms		
Days before injection	No. of patients	Recovery of 20% SRR/20-dB PTA
14 – 21 d	10	9 (90%)
21-28 d	3	1 (33%)
28 d or more	1	None
Total	14	10 (71,4 %)

SRR = speech recognition rate; PTA= tritonal mean.

### Recovery Related With the Intensity of Hearing Loss

Pearson's test found no statistically significant correlations between initial loss (initial PTA) and improvement after OCT (r = −0.115, p=0.696) (Fig. 1), and between initial loss and improvement after ITC (r = 0.134, p=0.647) (Fig. 2).

### Recovery at three moments (Initial, after OCT, and after ITC)

**Figure 4:**
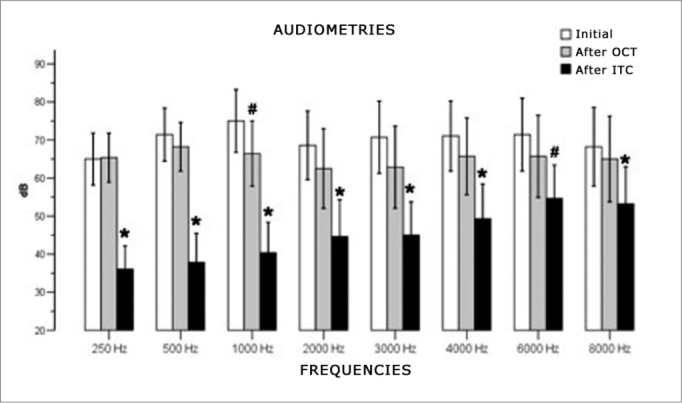
Comparison between recovery and types of treatment.

A comparison of audiometry measurement means in three moments (initial, after OCT and after ITC) at each frequency (250, 500, 1000, 2000, 3000, 4000, 6000, 8000 Hz) revealed statistically significant differences between measurements at all frequencies (Fig. 4).

At 250 Hz (F2, 26= 23.863, p<0.001) the audiometry value after ITC was significantly lower compared to the initial value (p<0.001) and after OCT (p<0.001). At 500 Hz (F2, 26=32.234, p<0.001), the audiometry value after ITC was lower than the initial value (p<0.001) and after OCT (p<0.001). At 1000 Hz (F2, 26=48.018, p<0.001), the mean audiometric value after ITC was statistically lower than the initial mean (p<0.001) and lower than the mean after OCT (p<0.001). There was also a statistically significant difference between audiometry after OCT and initial audiometry (p=0.027). At 2000 Hz (F2,26=16.558, p<0.001), the mean audiometry values after ITC were significantly lower compared to the initial values (p=0.001), and after OCT (p=0.003).

At 3000 Hz (F2,26=17.138, p<0.001), a statistically significant difference was found between initial audiometry values and after ITC (p<0.001), and between post ITC and post OCT values (p=0.007). At 4000 Hz (F2, 26=14.470, p<0.001) the mean audiometry values after ITC were significantly lower compared to initial values (p=0.002) and after OCT (p=0.011). At 6000 Hz (F2, 26=9.488, p=0.001), the audiometry value after ITC was lower than the initial value (p=0.004), and there was no difference between values after ITC and after OCT (p=0.066).

At 8000 Hz (F2, 26=9.715, p=0.001), a statistically significant difference was found between initial audiometry values and after ITC (p=0.011) and between post ITC and post OCT values (p=0.019).

**Figure 5:**
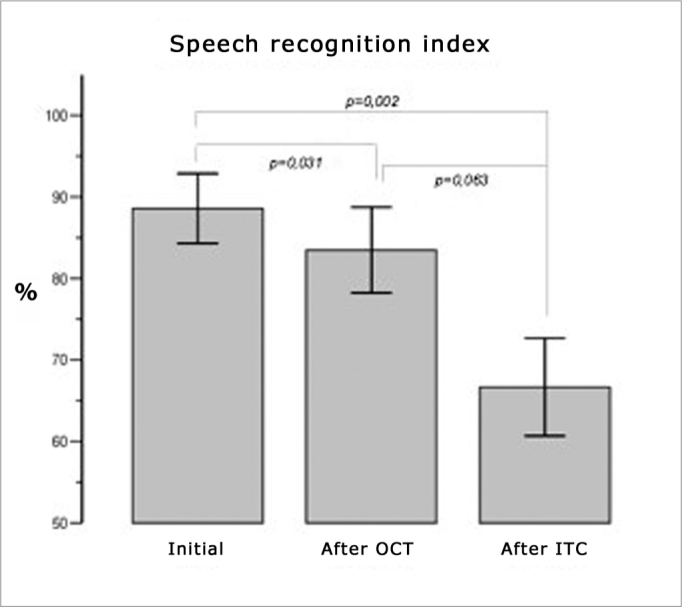
Comparison between recovery of the SRR after oral and intratympanic corticosteroid therapy

There was a statistically significant difference between SRR values in three audiometry tests (F2, 26=13.208, p<0.001). The multiple comparisons procedure showed that these differences were present in initial measurements, after ITC (p=0.002) and between initial and after OCT value (p=0.031) (Fig. 5).

## DISCUSSION

Wilson et al. conducted a double-blind placebo-controlled study that demonstrated a statistically significant benefit of systemic corticosteroids for hearing recovery in patients with sudden sensorineural hearing loss (SSHL).^1^ Other studies have also shown the benefit of systemic corticosteroid therapy for hearing loss recovery in SSHL patients.^3^^,^^5^^,^^6^^,^^7^^,^^11^ A few researchers, however, have published discouraging results as to the benefits of systemic corticosteroids compared to placebo.^2^^,^^4^^,^^8^

The precise mechanism by which steroids may improve hearing remains unknown; both glucocorticoid and mineralocorticoid receptors may be found in the inner ear.^28^ A few studies have shown that the main roles of steroids in the treatment of SHL are to protect the cochlea from the harmful effects of inflammatory mediators such as the tumor necrosis factor (TNF-a and NFK-b), which is elevated in infection and inflammation,^29^ to increase cochlear blood flow^30^ thereby avoiding cochlear ischemia,^31^ to avoid noise-induced hearing loss,^32^ and to regulate protein synthesis in the inner ear.^33^ Studies have shown that the vascular stria, which regulates Na/K secretion for maintaining the endocochlear potential, is a site of injury in SHL.^34^ Systemic corticosteroid therapy improves vascular stria function and may preserve its morphology,^35^ and therefore its potential for recovering from SHL.

Several studies have shown that intratympanic corticosteroids are safe and do not appear to cause any histological changes.^12^^,^^36^, ^37^, ^38^, ^39^, ^40^

It has been demonstrated that intratympanic corticosteroids increase cochlear blood flow,^12^^,^^36^ prevents aminoglycoside toxicity^41^ and improves ionic homeostasis, which is necessary for adequate cochlear function.^39^ Intratympanic corticosteroids protect the vascular stria in otitis media.^42^ Studies of patients with tinnitus have shown that intratympanic dexamethasone is not effective for relieving symptoms, but has no adverse effects against cochlear function, as evidences in otoacoustic emissions.^38^^,^^39^ Other studies have also suggested that intratympanic corticosteroids might not be beneficial in the treatment of hearing loss. These authors suspected that there was a potential risk of decreased cochlear function because of intratympanic therapy.^43^ Intratympanic therapy has also been charged with causing inflammation to reach the round window.^44^ Yang et al. argued that intratympanic therapy was ineffective for preventing immune mediated labyrinthitis induced by the KLH factor in guinea-pigs, and therefore would be ineffective in the treatment of SHL.^45^

### Cochlear Pharmacokinetics

Steroids administered intratympanically may attain high concentrations in the perilymph, higher than when administered intravenously or orally.^13^^,^^46^^,^^47^ Using markers such as phenyl-ammonia (TMPA)^48^ and peroxidase,^49^ researchers have demonstrated a non-uniform distribution, where higher concentrations were reached close to the round window (basal gyrus) compared to the apical gyri. Salt showed that substances could reach the vestibule by means of extracellular pathways between scalae and through the spiral ligament.^50^^,^^51^ These studies and those of Parnes^13^ suggest that there is non-linear flow and interscala pathways for substances administered intratympanically.^13^^,^^50^, ^51^, ^52^

### Studies on Intratympanic corticosteroids for the Treatment of SSHL

Silverstein (1996) published the first report of intratympanic corticosteroid use for the treatment of SSHL,12 followed by Parnes (1999).^13^ Several other researchers have published their results, mostly after 2001.^9^^,^^14^, ^15^, ^16^, ^17^, ^18^, ^19^, ^20^, ^21^^,^^23^, ^24^, ^25^, ^26^, ^27^ Many of these papers reported on the benefits of intratympanic corticosteroids for the treatment of SSHL in patients where systemic therapy failed.

Recent papers have described studies in which patients were given intratympanic corticosteroids as the therapy of choice or as adjuvant therapy with oral corticosteroid therapy.^23^^,^^24^ In most of these studies, the addition of intratympanic therapy to oral therapy had no significant effect on the recovery of hearing. Lauterman^23^ compared patients that were given intratympanic corticosteroids and systemic corticosteroid therapy with another group treated only with oral corticosteroid therapy and found no benefit from adding intratympanic corticosteroids. Battista^24^ reported a minor improvement following intratympanic corticosteroids in SHL patients in a study of 25 patients with profound sensorineural hearing loss (90-dB PTA).

Lefebre and Staeker treated 6 SHL with methylprednisolone infused through a microcatheter during 8 to 10 days. Systemic treatment had failed in all of these patients. The tone thresholds of six patients improved on average from 16.25 to 25 dB.^16^ Kopke et al. reported on the result of intratympanic microcatheters therapy (62.4 mg/ mL methylprednisolone at 10 ml/hour for 14 days) in the treatment of SHL patients; in this study, patients had been treated before with oral prednisolone for 2 weeks without success. Five of six treated patients showed improved hearing, four of which returned to normal hearing status.^17^

Gouveris treated 40 patients with intratympanic corticosteroids in a prospective study of patients in which systemic therapy had been unsuccessful. Efficacy was lower in patients with profound hearing loss or high-frequency loss.^19^

Lauterman et al. (2005) reported the results of a prospective study in which a group of SHL patients was treated with intratympanic methylprednisolone as the first treatment, and was then compared with another group undergoing systemic therapy (rheological agents and prednisolone). There was no difference in the efficacy of both treatments.

Ho et al. (2005) published a randomized placebo-controlled study of 39 SHL patients in which 29 (74%) did not benefit from systemic therapy, and were then randomized into two groups; fifteen patients were given intratympanic corticosteroids and 14 were given systemic therapy. The recovery rate of hearing in the intratympanic therapy group was 53%, while it was 7.1% in patients given systemic therapy; the improvement criterion was a 30 dB gain in PTA. Age, delayed beginning of therapy and sex did not alter the response to treatment.^20^

Battista (2005) treated 25 patients diagnosed with profound sensorineural hearing loss; systemic and intratympanic therapy was given jointly (adjuvant therapy). Results were generally poor: only 12% (3 of 25) had complete or partial recovery of hearing.^24^

Slattery et al. published a study of 20 SHL patients treated unsuccessfully with systemic therapy, and then treated with intratympanic methylprednisolone. A 10dB improvement - PTA or 12% discrimination - was seen in 55% of patients, as well as decreased tinnitus.^9^

Dallan et al. (2006) treated 8 patients with intratympanic methylprednisolone in a prospective study; there was a 75% improvement rate after a single injection.^26^

Choung et al. reported a 38% improvement rate in a group of patients treated with intratympanic and systemic therapy; another group treated only with systemic therapy had a 6.1% improvement rate.^25^

As the natural history of SHL suggest high recovery rates, it is rather difficult to establish whether interventions really increase those rates. The natural history of untreated SHL patients has recovery rates ranging from 31% to 65%.^1^^,^^3^^,^^4^^,^^8^ Several reasons may explain these different published rates; the best one may be the possibility that each author measures successful recovery differently.

A review of studies published to this date shows that the definition of success or post-therapy improvement may differ significantly between authors. There are no established criteria for defining recovery in SHL patients, especially in those cases of secondary recovery after failure of the first treatment. Recovery criteria may range from any improvement in PTA or SRR,14 a 10 dB improvement in the PTA or 10% in the SRR,15 or even Wilson et al.'s criterion,1 which defines improvement as a 50% recovery of the initial loss.^24^

A metanalysis of the literature would be complicated by the huge variation in protocols and data presentation of patients. If we apply similar improvement criteria in most of these studies, however, one would probably find similar recovery rates.

Our recovery rate (about 70%) is similar to that in other studies that applied the same criteria for defining improvement, the number of injections, their concentration and the type of steroid.

Shaia and Sheehy noted a significant improvement in patients treated within a week of the onset of hearing loss. However, some patients that started treatment after 3 months also recovered (10%).^53^ Fuse et al. found that most patients that recovered completely after OCT improved within 7 to 10 days after starting steroid medication. A longer term follow-up of patients (3 months to 2 years) showed that none of the patients that did not recover in the short term returned to normal hearing status. These authors found that corticosteroid-resistant patients continued to recover poorly in a long term follow-up.^6^

Ito et al. assessed 90 SHL patients and found that patients that recovered within 2 weeks had more chances of recovering more completely. Patients that recovered poorly within 2 weeks had insignificant further improvement in longer than 1 month monitoring.^10^

Lefebre reported that 100% of sensorineural SHL patients treated with corticosteroids recovered within 7 days.^16^

We excluded patients with Ménière's disease and fluctuating hearing loss because of the difficulty of separating treatment as a defining factor for recovery. Contralateral normal hearing was present in 92.82% of patients.

Three patients had diabetes mellitus; the recovery rate in these patients was similar to non-diabetic subjects. There were no complications in this group of patients; all three (21.42%) had type 2 diabetes mellitus and underwent systemic 1mg/kg/day prednisolone therapy with the approval of their assisting physicians.

Chandrasekhar found that 3 of 3 diabetic SHL patients improved with intratympanic therapy,14 which runs opposite to the general feeling that diabetic SHL patients would fare worse than non-diabetic subjects.^53^^,^^54^

Patients with vertigo in our study had lower recovery rates compared to patients without vertigo. The presence of vertigo has been associated with a worse prognosis in several studies.^2^^,^^4^^,^^21^^,^^52^^,^^53^

Patients with profound hearing loss had similar recovery rates to patients with losses below 90 dB. Pearson's test showed that there were no statistically significant correlations between initial loss (initial PTA) and improvement after OCT (r = −0.115, p=0.696) (Fig. 1), and between initial loss and improvement after ITC (r = 0.134, p=0.647) (Fig. 2). Several studies, however, have suggested that patients with profound hearing loss have a worse prognosis.^2^^,^^7^^,^^11^^,^^24^

The PTA of only one patient worsened (defined as any worsening in follow-up exams) after injections. Only this patient had a mildly worse SRR (defined as any worsening in follow-up exams) after injections. This may be explained in part by the fact that this patient had temporary perforation with otorrhea.

No other patient had perforation, otitis media, otorrhea, otalgia, or vertigo after injections. No other complications were reported. We reduced partly the limitations of this study by choosing a prospective study; it did not, however, contain a control group to compare the results of treated and untreated patients. This was because all available patients for therapy - after unsuccessful systemic therapy - chose to enter the study; we found it unethical to block access to Intratympanic therapy.

Although we were aware that dexamethasone diffuses well through the round window, we chose methylprednisolone because Parnes demonstrated that this drug has a higher concentration and remains longer in the perilymph after intratympanic administration compared to hydrocortisone or dexamethasone.^13^

Finally, we opted for three injections based on the reflections of other authors who ended their studies unsatisfied with single injection therapy and recommended continuous infusion or multiple injections in subsequent studies.^16^^,^^17^

## CONCLUSION

The recovery rate in this group of patients undergoing salvage treatment (after systemic therapy had failed) was 71.4%; this was a 20 dB improvement in the tritonal mean or 20% in the SRR. This study included only patients in whom systemic therapy had failed, and showed that three intratympanic methylprednisolone injections raised the tone thresholds and SRR in this group of patients with SSHL that did not benefit from oral corticosteroid therapy, evidence that this novel technique is a modern alternative for the treatment of patients with SSHL.

## References

[1] Wilson WR, Byl FM, Laird N. (1980). The efficacy of steroids in the treatment of idiopathic sudden hearing loss. A double- blind clinical study. Arch Otolaryngol..

[2] Byl FM (1984). Sudden hearing loss: eight years' experience and suggested prognostic table. Laryngoscope..

[3] Chen CY, Halpin C, Rauch SD. (2003). Oral steroid treatment of sudden sensorineural hearing loss: a ten year retrospective analysis. Otol Neurotol..

[4] Mattox DE, Simmons FB. (1977). Natural history of sudden sensorineural hearing loss. Ann Otol Rhinol Laryngol..

[5] Zadeh MH, Storper IS, Spitzer JB. (2003). Diagnosis and treatment of sudden-onset sensorineural hearing loss: a study of 51 patients. Otolaryngol Head Neck Surg..

[6] Fuse T, Aoyagi M, Funakubo T, Sakakibara A, Yoshida S. (2002). Shortterm outcome and prognosis of acute low-tone sensorineural hearing loss by administration of steroid. ORL J Otorhinolaryngol Relat Spec..

[7] Slattery WH, Fisher LM, Iqbal Z, Liu N. (2005). Oral steroid regimens for idiopathic sudden sensorineural hearing loss. Otolaryngol Head Neck Surg..

[8] Cinamon U, Bendet E, Kronenberg J. (2001). Steroids, carbogen or placebo for sudden hearing loss: a prospective double-blind study. Eur Arch Otorhinolaryngol..

[9] Slattery WH, Fisher LM, Iqbal Z, Friedman RA, Liu N. (2005). Intra-tympanic steroid for the treatment of sudden hearing loss. Otolaryngol Head Neck Surg..

[10] Ito S, Fuse T, Yokota M, Watanabe T, Inamura K, Gon S (2002). Prognosis is predicted by early hearing improvement in patients with idiopathic sudden sensorineural hearing loss. Clin Otolaryngol..

[11] Moskowitz D, Lee KJ, Smith HW. (1984). Steroid use in idiopathic sudden sensorineural hearing loss. Laryngoscope..

[12] Silverstein H, Choo D, Rosenberg SI, Kuhn J, Seidman M, Stein I. (1996). Intratympanic steroid treatment of inner ear disease and tinnitus (preliminary report). Ear Nose Throat J..

[13] Parnes LS, Sun AH, Freeman DJ. (1999). Corticosteroid pharmacokinetics in the inner ear fluids: an animal study followed by clinical application. Laryngoscope..

[14] Chandrasekhar SS. (2001). Intratympanic dexamethasone for sudden sensorineural hearing loss: clinical and laboratory evaluation. Otol Neurotol..

[15] Gianoli GJ, Li JC. (2001). Transtympanic steroids for treatment of sudden hearing loss. Otolaryngol Head Neck Surg..

[16] Lefebvre PP, Staecker H. (2002). Steroid perfusion of the inner ear for sudden sensorineural hearing loss after failure of conventional therapy: a pilot study. Acta Otolaryngol..

[17] Kopke RD, Hoffer ME, Wester D, O'Leary MJ, Jackson RL. (2001). Targeted topical steroid therapy in sudden sensorineural hearing loss. Otol Neurotol..

[18] Jackson LE, Silverstein H. (2002). Chemical perfusion of the inner ear. Otolaryngol Clin North Am..

[19] Gouveris H, Selivanova O, Mann W. (2005). Intratympanic dexamethasone with hyaluronic acid in the treatment of idiopathic sudden sensorineural hearing intravenous steroid and vasoactive therapy. Eur Arch Oto-rhinolaryngol..

[20] Ho GM, Lin HG, Shu MT. (2004). Effectiveness of intratympanic dexamethasone injection in sudden deafness patients as salvage treatment. Laryngoscope..

[21] Herr BD, Marzo SJ. (2005). Intratympanic steroid perfusion for refractory sudden sensorineural hearing loss. Otolaryngol Head Neck Surg..

[22] Itoh A, Sakata E. (1991). Treatment of vestibular disorders. Acta Otolaryngol Suppl..

[23] Lauterman J, Sudhoff H, Junker R. (2005). Transtympanic corticoid therapy for acute profound loss. Eur Arch Otorhinolaryngol..

[24] Battista RA. (2005). Intratympanic dexamethasone for profound idiopathic sudden sensorineural hearing loss. Otolaryngol Head Neck Surg..

[25] Choung YH, Park K, Shin YR, Cho MJ. (2006). Intratympanic dexamethasone injection for refractory sudden sensorineural hearing loss. Laryngoscope..

[26] Dallan I, Bruschini P, Nacci A. (2006). Transtympanic steroids as a salvage therapy in sudden hearing loss: preliminary results. ORL J Otorhinolaryngol Relat Spec..

[27] Xenellis J, Papadimitriou N, Nikolopoulos T, Maragoudakis P, Segas J, Tzagaroulakis A (2006). Intratympanic steroid treatment in idiopathic sudden sensorineural hearing loss: a control study. Otolaryngol Head Neck Surg..

[28] Rarey KE, Luttge WG. (1989). Presence of type I and type 2/IB receptors for adrenocorticosteroid hormones in the inner ear. Hear Res..

[29] Stockroos RJ, Albers FW, Schirm J. (1998). The etiology of idiopathic sudden sensorineural hearing loss. Experimental herpes simplex virus infection of the inner ear. Am J Otol..

[30] Nagura M, Iwasaki S, Wu R, Mizuta K, Umemura K, Hoshino T. (1999). Effects of corticosteroid, contrast medium and ATP on focal microcirculatory disorders of the cochlea. Eur J Pharmacol..

[31] Tabuchi K, Oikawa K, Uemaetomari I, Tsuji S, Wada T, Hara A. (2003). Glucocorticoids and dehydroepiandrosterone sulfate ameliorate ischemia-induced injury of the cochlea. Hear Res..

[32] Lamm K, Arnold W. (1998). The effect of prednisolone and non-steroidal anti-inflammatory agents on the normal and noise- damaged guinea pig inner ear. Hear Res..

[33] Yao X, Buhi WC, Alvarez IM, Curtis LM, Rarey KE. (1995). De novo synthesis of glucocorticoid hormone regulated inner ear proteins in rats. Hear Res..

[34] Lin DW, Trune DR. (1997). Breakdown of stria vascularis blood- labyrinth barrier in C3H/lpr autoimmune disease mice. Otolaryngol Head Neck Surg..

[35] Trune DR, Wobig RJ, Kempton JB, Hefeneider SH. (1999). Steroid treatment improves cochlear function in the MRL.MpJ- Fas(lpr) autoimmune mouse. Hear Res..

[36] Shirwany NA, Seidman MD, Tang W. (1998). Effect of transtympanic injection of steroids on cochlear blood flow, auditory sensitivity, and histology in the guinea pig. Am J Otol..

[37] El-Hennawi DM, El-Deen MHB, Abou-Halawa AS, Nadeem HS, Ahmed MR. (2005). Efficacy of intratympanic methylpred- nisolone acetate in treatment of drill-induced sensorineural hearing loss in guinea pigs. J Laryngol Otol..

[38] Yilmaz I, Yilmazer, Erkan AN, Aslan SG, Ozluoglu LN. (2005). Intratympanic dexamethasone injection effects on transient- evoked otoacoustic emission. Am J Otololaryngol..

[39] Araujo MFS, Oliveira CA, Bahmad F (2005). Intratympanic Dexamethasone Injections as a Treatment for Severe, Disabling Tinnitus. Does it work?. Arch Otolaryngol..

[40] Fukushima M, Kitahara T, Uno Y, Fuse Y, Doi K, Kubo T. (2002). Effects of intratympanic injection of steroids on changes in rat inner ear aquaporin expression. Acta Otolaryngol..

[41] Himeno C, Komeda M, Izumikawa M, Takemura K, Yagi M, Weiping Y (2002). Intra-cochlear administration of dexamethasone attenuates aminoglyco- side ototoxicity in the guinea pig. Hear Res..

[42] Sone M, Hayashi H, Yamamoto H, Tominaga M, Nakashima T. (2003). A comparative study of intratympanic steroid and NO synthase inhibitor for treatment of cochlear lateral wall damage due to acute otitis media. Eur J Pharmacol..

[43] Spandow O, Hellstrom S, Anniko M. (1988). Impaired hearing following instillation of hydrocortisone into the middle ear. Preliminary report from an animal study. Acta Otolaryngol Suppl..

[44] Nordang L, Linder B, Anniko M. (2003). Morphologic changes in round window membrane after topical hydrocortisone and dexamethasone treatment. Otol Neurotol..

[45] Yang GSY, Song HT, Keithley EM, Harris JP. (2000). Intratympanic immunosuppressives for prevention of immunemediated sensorineural hearing loss. Am J Otol..

[46] Chandrasekhar SS, Rubinstein RY, Kwartler JA, Gatz M, Connelly PE, Huang E (2000). Dexamethasone pharmacokinetics in the inner ear: comparison of route of administration and use of facilitating agents. Otolaryngol Head Neck Surg..

[47] Bachmann G, Su J, Zumegen C, Wittekindt C, Michel O. (2001). Permeabilitat derrunden Fenster membran fur Prednisolon-21-Hydrogensuccinat. Prednisolongehalt der Perilymphe nach lokaler Applikation vs. systemischer Injektion. HNO..

[48] Salt AN, Ma Y. (2001). Quantification of solute entry into cochlear perilymph through the round window membrane. Hear Res..

[49] Saijo S, Kimura RS. (1984). Distribution of HRP in the inner ear after injection into the middle ear cavity. Acta Otolaryngol..

[50] Salt AN, Ohyama K, Thalmann R. (1991). Radial communication between the perilymphatic scalae of the cochlea. I: Estimation by tracer perfusion. Hear Res..

[51] Salt AN, Ohyama K, Thalmann R. (1991). Radial communication between the perilymphatic scalae of the cochlea. 2: Estimation by bolus injection of tracer into the sealed cochlea. Hear Res..

[52] Plontke S, Zenner HP. (2002). Pharmacokinetic considerations in intratympanic drug delivery to the inner ear. Acta Otorhinolaryngol Belg..

[53] Shaia FT, Sheehy J. (1976). Sudden sensorineural hearing impairment: a report of 1220 cases. Laryngoscope..

[54] Fukui M, Kitagawa Y, Nakamura N, Kadono M, Mogami S, Ohnishi M (2004). Idiopathic sudden hearing loss in patients with type 2 diabetes. Diabetes Res Clin Pract..

